# Robotic stapled cardioplasty, an alternative before esophagectomy

**DOI:** 10.1007/s13304-025-02161-w

**Published:** 2025-03-19

**Authors:** Elisenda Garsot, Arantxa Clavell, Pau Moreno, Marta Viciano

**Affiliations:** 1 Department of Surgery, Faculty of Medicine, Universitat Autonoma de Barcelona, Campus UAB, Bellaterra, 08913 Barcelona, Spain; 2 Department of General and Digestive Surgery, Hospital Universitari Germans Trias I Pujol, Carretera del Canyet s/n, Badalona, 08916 Barcelona, Spain

**Keywords:** Achalasia, Robotic, Cardioplasty, End stage, Revision surgery

## Abstract

Robotic stapled cardioplasty offers a novel, minimally invasive alternative for treating end-stage achalasia, a rare esophageal motor disorder. Achalasia is characterized by the inability of the lower esophageal sphincter to relax and reduced peristalsis, with about 5% of patients progressing to a stage where esophagectomy is often the only treatment option. This study introduces robotic stapled cardioplasty as a less aggressive, organ-preserving approach. Using a robotic platform, the procedure improves visibility, precision, and careful dissection, making it especially useful in complex revision cases. To our knowledge, this is the first reported case of robotic stapled cardioplasty for end-stage achalasia. It appears to be a safe and effective minimally invasive alternative to esophagectomy and may also serve as a rescue procedure for patients who have exhausted other treatment options before considering organ resection.

**Graphical Abstract:**

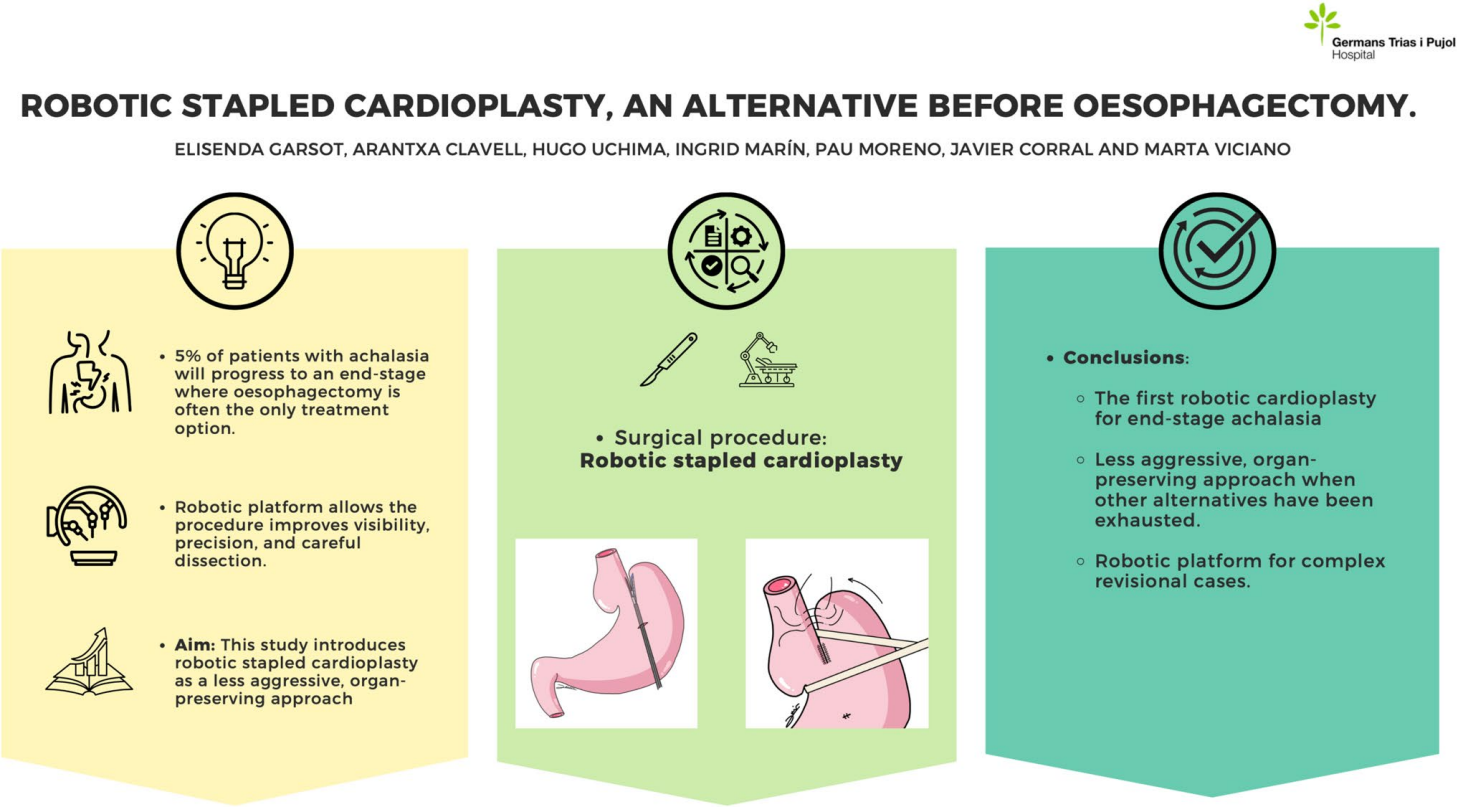

**Supplementary Information:**

The online version contains supplementary material available at 10.1007/s13304-025-02161-w.

## Introduction

Achalasia is a rare primary motility disorder of the esophagus characterized by aperistalsis and incomplete relaxation of the lower esophageal sphincter (LES) that affects up to 3 patients out of every 100,000. Dysphagia is present in most patients, often accompanied by regurgitation of undigested food, and the primary goal of treatment is to improve the quality of life, as current options cannot restore native peristalsis. The primary treatments of choice are pneumatic dilation (PD), Heller’s myotomy (LHM), and peroral endoscopic myotomy (POEM). However, in 10–15% of patients, esophageal function will deteriorate over time, and up to 5% will develop what is commonly called end-stage achalasia [1].

The definition of end-stage achalasia is not straightforward, but the International Society for Diseases of the Esophagus (ISDE) recommends barium esophagram as the defining study, characterized by massive dilation and tortuosity, known as sigmoid esophagus [2]. In the case of end-stage achalasia, multiple techniques have been described to try to improve patient symptoms, including endoscopic treatments (pneumatic dilation, botulinum toxin, POEM, Peroral Plication of the Esophagus (POPE)), LHM, cardioplasty, cardiotomy with various types of reconstruction, and esophageal resection as the most radical option [1]. However, it is not clear what the treatment of choice is or when it should be applied. Intermediate situations also exist where patients are not in the final phase of the pathology but have exhausted the most effective alternatives available: LHM, POEM, or pneumatic dilation.

We present a rarely used technique, cardioplasty, of which only a few cases are described in the literature, as an alternative for patients who no longer have other options before considering organ resection. In this case, it is performed with a robotic approach. The robot is especially indicated in revision cases in which greater precision is necessary to identify previously altered anatomical structures and avoid major injuries. Video support is provided to show the details of the technique with better precision.

To date, no robotic procedure of these characteristics has been described.

## Methods

### Case report

We present the case of a 49-year-old woman diagnosed with type II achalasia who was treated with LHM 9 years ago with good clinical results, but with subsequent symptomatic recurrence, so a POEM was performed 4 years later. She was currently experiencing a recurrence of symptoms with an Eckardt score of 6, thus requiring several pneumatic dilations.

**Fig. 1:**
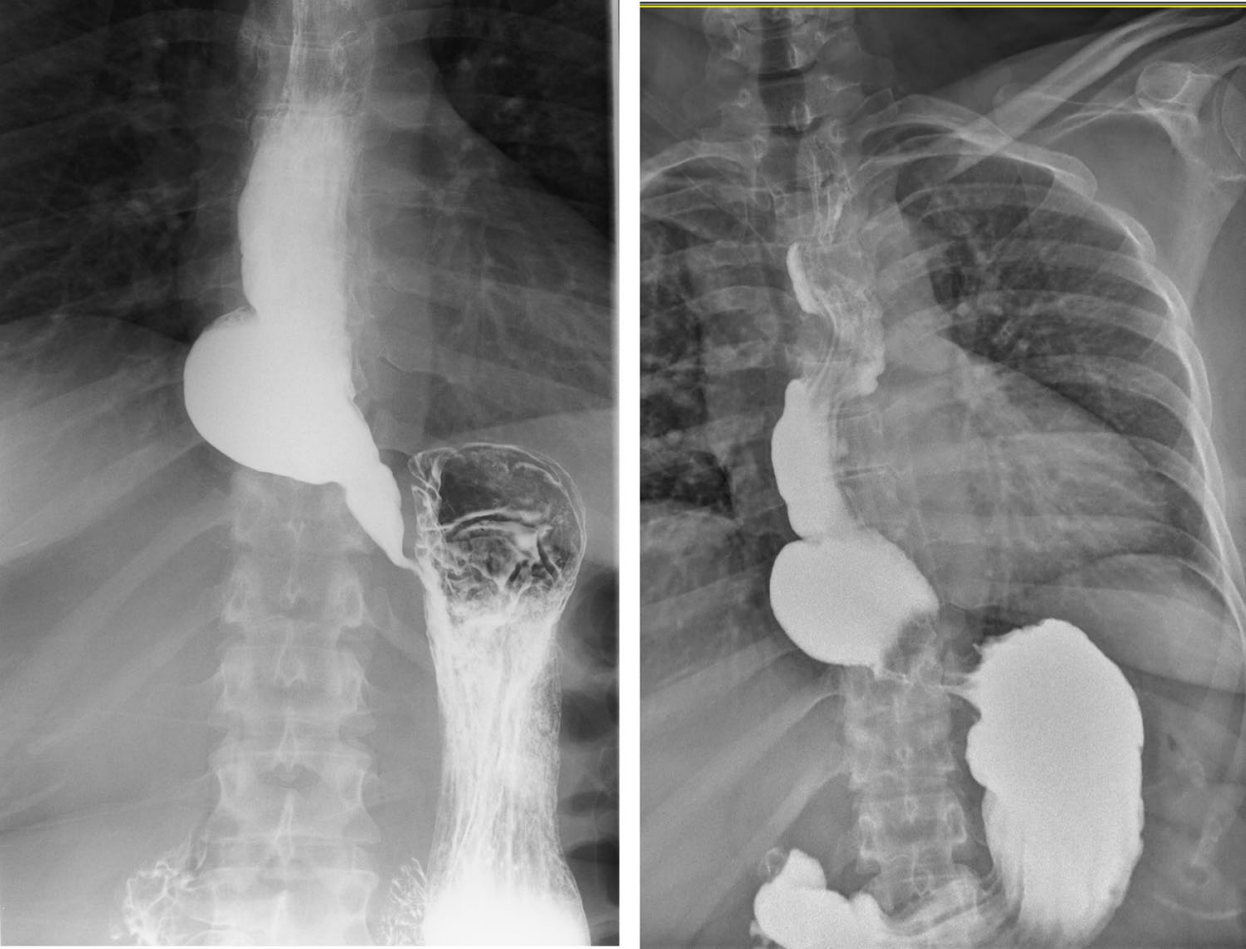
Contrast swallow before and after intervention

The diagnosis was assessed using gastroscopy, barium swallow, 24 h pH monitoring, and high-resolution manometry. The barium swallow showed a deep esophagus with a distal dilation resembling a sump or diverticulum, but not a clear sigmoid esophagus (Fig. 1). It was decided to perform a robotic cardioplasty for three reasons: (1) to provide a less aggressive treatment than an esophagectomy, (2) two previous myotomy treatments (laparoscopic and endoscopic), indicating that other conventional options had been attempted, and (3) a significant sump formation (and poor esophageal emptying) in the lower esophagus, demonstrated on barium swallow.

## Description of technique: robotic stapled cardioplasty

### Operating room and robot set up 

Da Vinci Xi or X platform (Intuitive Surgical Inc., Sunnyvale, CA) was used. The patient was placed in a supine position with arms extended on both sides of the body and in an inverted 45º Trendelenburg position. The robot was docked from the patient’s left side. The trocars were placed in the following positions in a linear fashion: one 8 mm trocar at the supraumbilical level, two 10 mm and 8 mm ports 8 cm apart to the left of the midline, and one 8-mm port to the left of the midline and right of the midline. A Nathanson liver retractor (Cook Medical, Dublin, Ireland) was used to hold the left lobe of the liver, which was introduced through a 5 mm hole at the subxiphoid level.

### Surgical procedure 

**Fig. 2:**
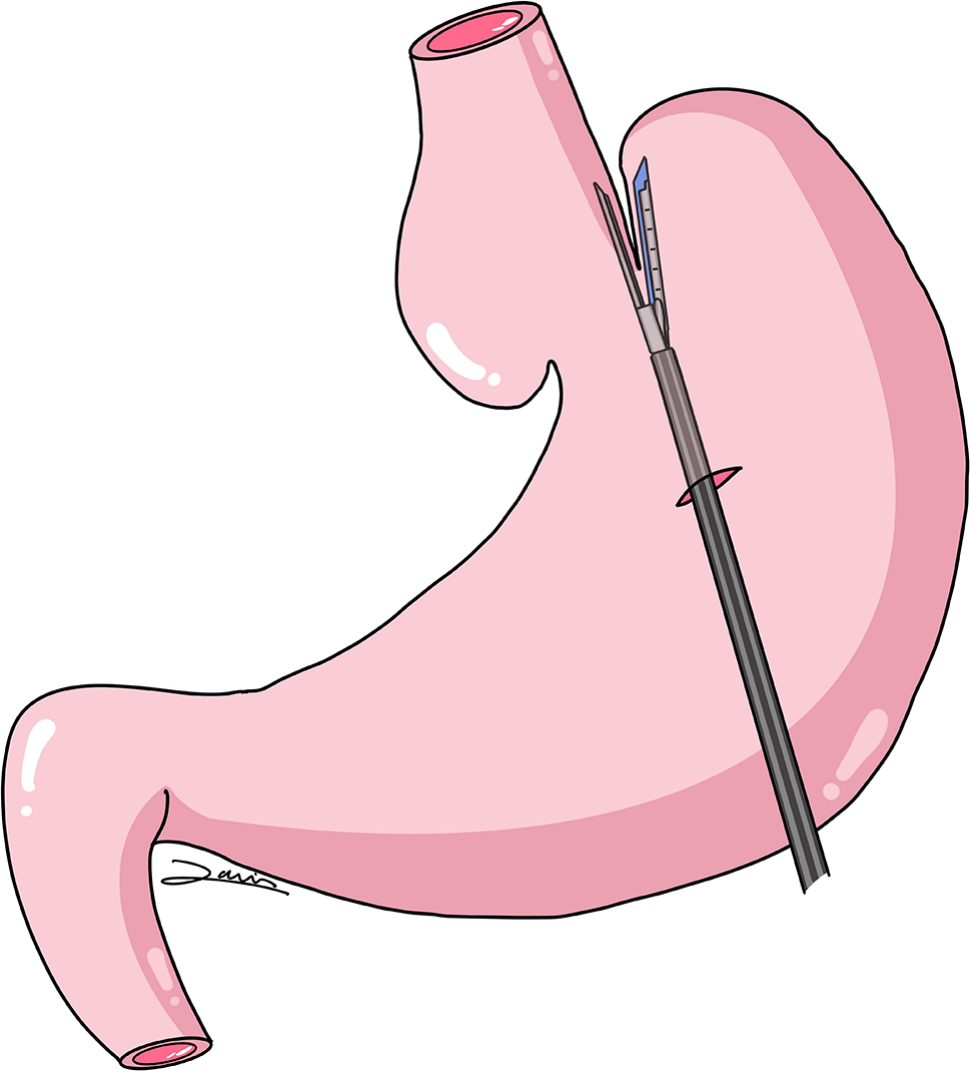
Stappled cardioplasty

**Fig. 3:**
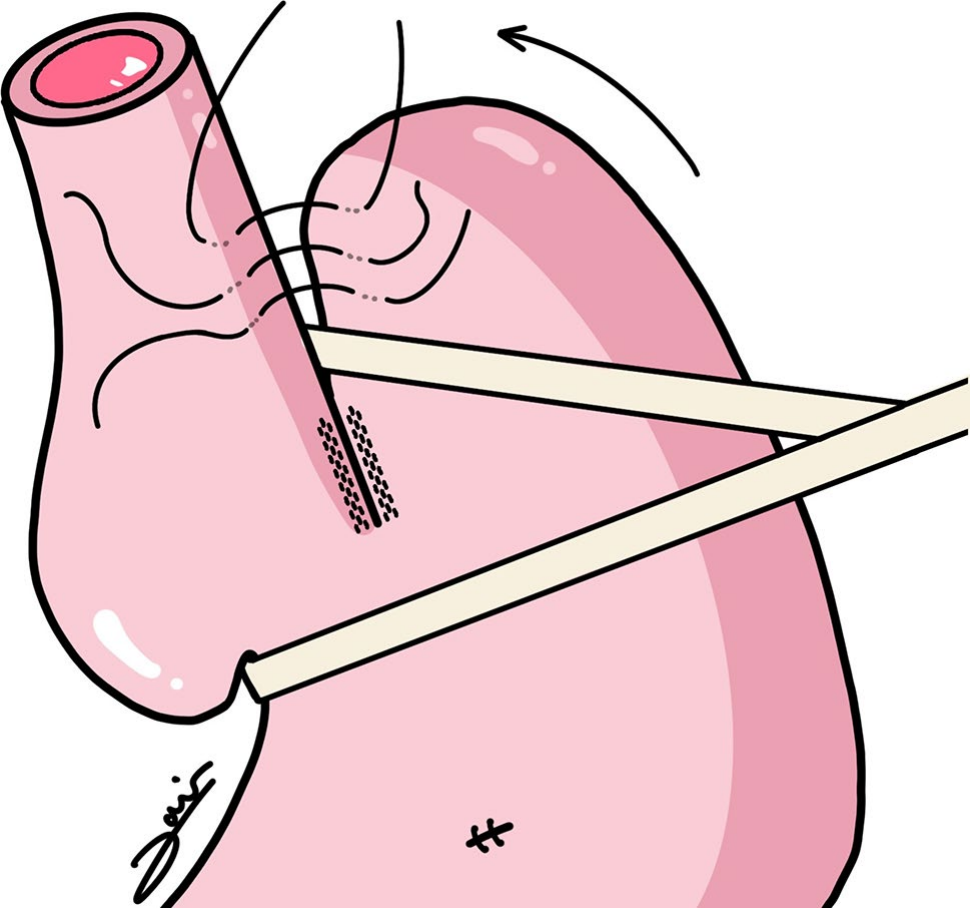
Lortat-Jacob anti-reflux procedure

The first step involved the anatomical dissection of the esophageal hiatus area and the gastroesophageal junction (GEJ), dismantling the previous Dor fundoplication and identifying the different structures. During the dissection, there was a small perforation of the exposed mucosa (around the previous myotomy), which was promptly identified and repaired with a simple suture. The technique performed involved making a gastrotomy in the proximal stomach, passing a 60 mm stapling device into the stomach, placing one jaw in the distal esophagus across the GEJ and the other jaw in the gastric fundus juxtaposed to the left of the distal esophagus (Fig. 2). The stapler was fired, creating an esophagogastrostomy. The gastrotomy was closed with a running barbed suture. An intraoperative fibrogastroscopy was performed concurrently with the procedure to facilitate the insertion of the two jaws of the endostapler and to evaluate the final outcome. Likewise, the simultaneous fibrogastroscopy allowed us to rule out performing a Dor-type fundoplication, as placing the fundus in its final position would have resulted in the closure of the GEJ. In addition, the division of the short gastric vessels was avoided to preserve the option for a possible future gastroplasty. Therefore, a Lortat-Jacob anti-reflux procedure was ultimately performed (Fig. 3). See attached video (supplementary material).

### Preliminary results

The patient exhibited an appropriate postoperative recovery, beginning oral intake on the second postoperative day and being discharged on the fourth day. To date, with a brief follow-up of 24 months, the patient has shown significantly improved symptoms with and Eckardt score of 3 actualmente, continues treatment with proton pump inhibitors (PPI), and demonstrates proper gastric emptying on the contrast swallow test (Fig. 1).

### Discussion 

In patients diagnosed with achalasia who experience persistent dysphagia after surgery, the most common causes of myotomy failure include incomplete myotomy, fibrosis or scarring that reduces compliance at the lower esophageal sphincter (LES), and obstruction due to extrinsic compression from the fundoplication. A thorough evaluation of the cause of the persistent or recurrent symptoms is recommended before initiating further treatment [5].

In any case, in 5% of cases, what is known as end-stage achalasia develops, characterized by a recurrence of symptoms and diagnosed through an esophagram, which reveals a dilated and tortuous esophagus. In many of these cases, other procedures have already been exhausted, leaving us with limited treatment options and with esophagectomy being practically the only remaining option.

Different alternatives have been proposed for the treatment of end-stage achalasia. In most cases, esophagectomy is chosen [1], but other procedures have been described with the idea of reducing the aggressiveness of the procedure or as an intermediate technique before organ resection. Treatment options include pneumatic dilation, botulinum toxin injection, peroral endoscopic myotomy (POEM), redo myotomy, or even esophagectomy, which is considered a last resort when all conservative strategies have failed or when the disease has progressed to its end stage [3]. It is crucial to understand the cause of symptom recurrence to determine the appropriate treatment for each case, and this presents a significant challenge. Therefore, this decision should be made through consensus in a multidisciplinary context and variables such as age, previous interventions, the overall health of the patient, and institutional limitations must be thoroughly considered and discussed by the team prior to the intervention.

The choice of technique should be based on the underlying cause. In cases of incomplete myotomy, it may be indicated to extend the myotomy or perform a new myotomy, ideally through a different approach (POEM or LHM, depending on the initial technique used). Both procedures are complex and carry a higher morbidity rate compared to primary myotomy [4].

In cases of periesophageal scarring or reflux-induced stricture, the options are more conservative, and treatment often involves dilations. However, surgical revision may occasionally be required, which is usually limited to adhesiolysis [3].

In some cases, the cause of dysphagia is a tight fundoplication, which can be treated with dilations but often requires surgical revision.

Finally, in end-stage achalasia, which represents the final phase of the disease and is often associated with limited treatment options, particularly when these have already been exhausted, esophagectomy is typically recommended in most cases, provided the patient’s condition permits it. [1].

There is a lack of data regarding the appropriate type of treatment for each case and the optimal timing for its implementation. Careful decision-making regarding the method of a second/third intervention is crucial, and it is often wise to begin with the least invasive option.

Cardioplasty has been proposed as a less aggressive alternative to esophagectomy in advanced stages of the disease. The technique was initially described in the first half of the twentieth century with subsequent variants being reported later on Refs. [5, 6]. In the last decade, there have been reports of adapting this technique to a minimally invasive approach, incorporating manual or mechanical anastomosis [7, 8]. However, this technique has not previously been described using a robotic approach.

The development of the robotic platform has made it possible to perform highly demanding procedures with high precision and security. The robot appears to offer particular advantages in revision cases, where greater precision is required to identify previously altered anatomical structures and to prevent significant injuries. 

In the surgical treatment of achalasia in the early stages, it has been shown that the number of mucosal perforations decreases with robotic approach and, in any case, that if they occur, they rarely go unnoticed and can be repaired in the same surgical act [9]. Revisional myotomy is a difficult operation and is characterized by a higher complication rate, including distal esophageal perforation, esophageal leak, and fundoplication failure [6]. This data supports the consideration of the robotic approach as an optimal method for performing demanding revisional procedures, such as those presented in our case.

The video illustrates the precision provided by the robotic approach in dissecting and identifying structures, enabling highly complex revision surgery to be conducted with utmost safety. Moreover, the robotic platform provides the capability to integrate intraoperative endoscopy images, which are highly advantageous for accurately positioning the endostapler, assessing outcomes and aiding in decision-making.

On the other hand, the primary long-term issue of cardioplasty appears to be the presence of gastroesophageal reflux [10], which is not always controlled with PPIs and in some cases resulting in the need for an esophagectomy. For this reason, we consider it important, where feasible, to combine an anti-reflux procedure with the technique. In the presented case, it was initially considered to reconstruct the Dor fundoplication, but intraoperative endoscopy revealed a closure of the esophageal lumen when positioning the gastric fundus over the gastroesophageal junction (GEJ). This prompted a change in strategy, leading to the use of a Lortat-Jacob type anti-reflux method, which, despite not being the most effective, has acceptable medium-term results.

## Conclusion

Robotic cardioplasty is a viable alternative for patients with achalasia who have exhausted other treatment options. This technique offers precision and control, both of which are essential for complex esophageal surgeries. The video provides a detailed view of the procedure, highlighting its feasibility and potential benefits.

However, this study has limitations that prevent the technique from being considered a definitive option in such cases: it is based on a single patient, and follow-up is limited. More time is required to evaluate long-term outcomes, particularly regarding the resolution of dysphagia and the potential development of gastro-esophageal reflux. Further studies with larger sample sizes are necessary to validate the efficacy of this surgical technique as an alternative for end-stage achalasia.

## Supplementary Information

Below is the link to the electronic supplementary material.

Supplementary file1 (MP4 44825 KB)

## Data Availability

All data generated or analyzed during this study are included in this article. Further enquiries can be directed to the corresponding author.
